# NMDA receptor activation stimulates transcription-independent rapid wnt5a protein synthesis via the MAPK signaling pathway

**DOI:** 10.1186/1756-6606-5-1

**Published:** 2012-01-04

**Authors:** Yichen Li, Bei Li, Xianzi Wan, Wei Zhang, Ling Zhong, Shao-Jun Tang

**Affiliations:** 1 School of Pharmaceutical Sciences and Laboratory Animal Center of Sun Yat-Sen University, Guangzhen, P. R. China; 2 Department of Neuroscience and Cell Biology, University of Texas Medical Branch, Galveston, TX77555, USA

**Keywords:** activity-regulated protein synthesis, Wnt protein, mTOR signaling, MAPK signaling, NMDA receptors

## Abstract

Wnt proteins are emerging key regulators of the plasticity and functions of adult brains. However, the mechanisms by which the expression of Wnt proteins is regulated in neurons are unclear. Using cortical primary cultures, we show here that activation of NMDA receptors (NMDARs) induces rapid Wnt5a protein synthesis and secretion. This NMDAR-regulated Wnt5a synthesis does not require transcription and is a result of activity-dependent translation. We also show that NMDAR-regulated Wnt5a translation depends on MAPK signaling but not mTOR signaling. Our findings suggest that the synaptic activity of CNS neurons activates NMDARs, which in turn stimulate translation from stored Wnt5a mRNA via the MAPK signaling pathway.

## Background

Wnts are secreted glycoproteins that regulate cell morphologies and behaviors by stimulating complicate intracellular signaling cascades. Previous work has established that Wnt signaling controls many oncogenic and developmental processes [1,2]. More recent studies have revealed that Wnt signaling is critically involved in key processes of the formation and plasticity of the nervous system, including neurogenesis [3], axon guidance [4], dendritic development [5], synaptic differentiation [6] and plasticity [7,8]. Abnormalities of Wnt signaling are implicated in major brain disorders such as Alzheimer's disease [9-11], Parkinson's disease [12,13], schizophrenia [14,15], and drug abuse [16]. Wnt5a is member of the Wnt protein family and plays important roles in outgrowth, guidance and branching of axons [17,18]; genesis of dopaminergic neurons [19]; and formation and plasticity of both excitatory and inhibitory synapses [20-22]. Wnt5a administration was reported to improve specific pathological processes of Alzheimer's [11] and Parkinson's diseases in animal models [12].

Wnt proteins bind to receptors to activate the Wnt/β-catenin canonical pathway and β-catenin-independent non-canonical pathways, which include the planar cell polarity (PCP) pathway and the Wnt/calcium (Ca^2+^) pathway [2,23-26]. In the canonical pathway, Wnts (such as Wnt3a) inhibit glycogen synthase kinase 3β (GSK-3β) and consequently stabilize β-catenin to regulate transcription [1]. Wnt5a is a prototypic Wnt ligand that activates the non-canonical pathways [27,28]. The activation of the PCP pathway stimulates Rho GTPases and c-Jun N-terminal kinase (JNK) to regulate cell morphogenesis and movement [29], whereas the activation of the Wnt/Ca^2+ ^pathway causes Ca^2+ ^to activate protein kinase C (PKC) and calcium/calmodulin dependent protein kinase II (CaMKII) [30]. In neurons, Wnt secretion is intimately governed by synaptic activity, especially the activation of NMDA receptors (NMDAR) [7].

In contrast to the detailed understanding of the intracellular signaling cascades initiated by Wnts, little is known about the upstream mechanisms that control the synthesis of Wnt proteins. Wayman et al. recently showed that NMDAR activation stimulates CREB-mediated Wnt2 transcription [31].

We report here a mechanism that couples NMDAR activation to Wnt5a protein synthesis in primary cortical cultures. We observed that NMDAR activation elicited rapid increase and secretion of Wnt5a protein. This NMDAR-regulated Wnt5a protein increase was blocked by translational but not transcriptional inhibitors. In addition, mitogen-activated protein kinase (MAPK) but not mammalian target of rapamycin (mTOR) inhibitors abolished this Wnt5a synthesis. Our findings suggest that a NMDAR/MAPK pathway controls the activity-regulated translation of Wnt5a mRNA in cortical neurons.

## Results

### NMDA receptor (NMDAR) activation rapidly increases Wnt5a in cortical cultures

**Figure 1:**
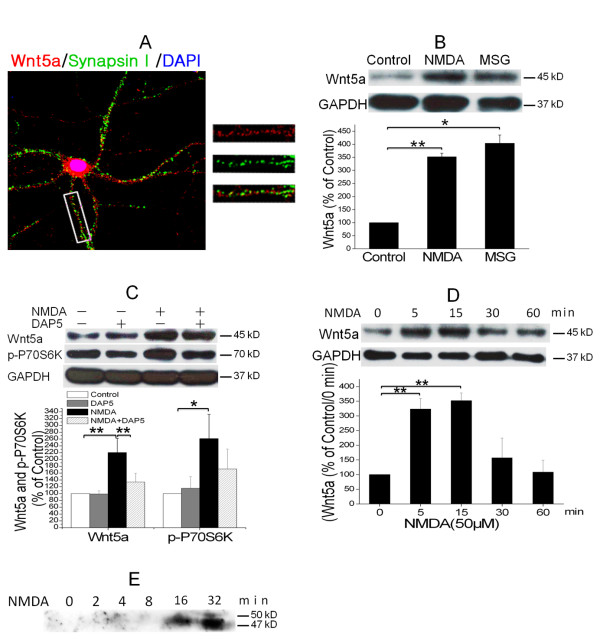
**NMDAR activation rapidly increases Wnt5a in cortical cultures**. A. Cellular localization of Wnt5a in neurons. Shown are confocal images of primary cortical neurons after double-fluorescent immunostaining with anti-Wnt5a (red) and anti-synapsin I (green) antibodies. The nucleus was stained by DAPI (blue). B. MSG or NMDA stimulation increased Wnt5a protein. Primary cortical neurons (10 DIV) were treated with 10 μΜ MSG or 50 μΜ NMDA for 15 min. Wnt5a protein was detected by Western blotting. Data in the summary graphs (mean ± SEM) were from three independent experiments (*, p < 0.05; **, p < 0.01; One-way ANOVA). C. NMDA receptor-regulated Wnt5a increase. Primary cortical neurons (10 DIV) were pre-treated with vehicle (Control) or 100 μΜ DAP5 for 30 min, then incubated with 50 μΜ NMDA for 15 min. Wnt5a and p-P70S6K(included as a marker for translation activation) were detected by Western blotting summarized in the graph (n = 3; *, p < 0.05; **, p < 0.01; One-way ANOVA). D. Dynamic expression of Wnt5a protein after NMDA stimulation. Primary cortical neurons (10 DIV) were treated with 50 μΜ NMDA for 0, 5, 15, 30 and 60 min followed by Western blotting analysis of Wnt5a (n = 3; *, P < 0.05; **, p < 0.01; One-way ANOVA). E. NMDA-induced Wnt5a protein secretion. Primary cortical neurons (10 DIV) were treated with 50 μΜ NMDA for 0, 2, 4, 8, 16 and 32 min, and Wnt5a protein in the media was concentrated and detected on immunoblots.

In an attempt to understand the regulation of Wnt5a expression by synaptic activity, we performed double-immunofluorescent staining of Wnt5a and synapsin I (a synaptic marker) to determine the cellular distribution of Wnt5a in mature cortical neurons (12 DIV). The specificity of the anti-Wnt5a antibody was confirmed with a Wnt5a knockout mouse. The results show that Wnt5a is localized in a somato-dendritic pattern (Figure 1A). In dendrites, Wnt5a is detected in regions adjacent to synapsin I signals, indicating a localization of Wnt5a nearby synapses. Next, we sought to determine whether Wnt5a protein expression is regulated by synaptic activity. Western blotting analysis of intracellular proteins indicated that glutamate stimulation (10 μM for 15 min) stimulation increased Wnt5a in cortical cultures by 4 fold (Figure 1B). Furthermore, NMDA stimulation (50 μM for 15 min) to activate NMDARs also increased Wnt5a protein by 3.5 fold (Figure 1B). The NMDA-induced Wnt5a increase was completely abolished by DAP5, a specific antagonist of NMDARs (Figure 1C), demonstrating that NMDA indeed elicited Wnt5a protein expression via the activation of NMDARs. These results indicate that NMDAR activation is sufficient to stimulate Wnt5a up-regulation. To characterize the kinetics of NMDAR-dependent Wnt5a protein expression, we determined the time course of NMDA stimulation. As shown in Figure 1D, Wnt5a protein was markedly increased within 5 min after NMDA administration. This observation suggested that NMDAR activation caused rapid Wnt5a synthesis. Strikingly, this increase of intracellular Wnt5a disappeared 30 min after NMDA stimulation (Figure 1D). Because NMDAR activation can evoke Wnt secretion (8), Wnt5a may be secreted to the medium after NMDA stimulation. To test this idea, we performed immunoblotting analysis of Wnt5a in culture media collected at 2, 4, 8, 16, or 32 min after NMDA stimulation. We observed that Wnt5a levels in media increased dramatically after 16 min (Figure 1E). This data indicates that NMDA activation increases not only the synthesis but also the secretion of Wnt5a. It appears that newly synthesized Wnt5a needs 8-16 min to complete the trafficking process for secretion.

### NMDAR-elicited Wnt5a increase requires translation but not transcription

**Figure 2:**
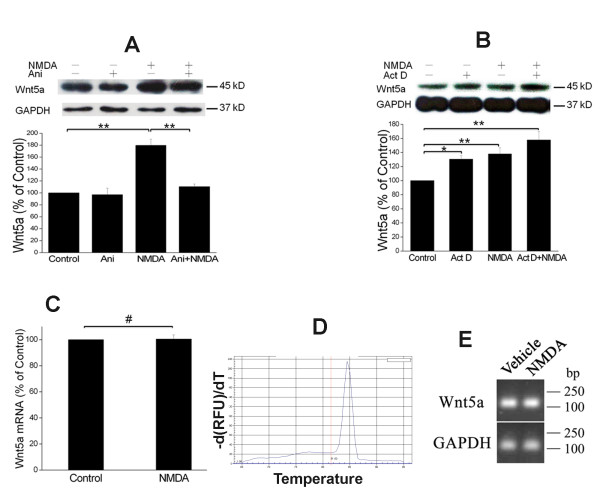
**NMDAR-elicited Wnt5a increase requires translation but not transcription**. A. Primary cortical neurons (10 DIV) were pre-treated with vehicle (Control) or 20 μΜ anisomycin for 30 min, and then incubated with 50 μΜ NMDA for 15 min, followed by Wnt5a immunoblotting. The Graph is a summary of three independent experiments (**, p < 0.01; One-way ANOVA). B. Primary cortical neurons (10 DIV) were pre-treated with vehicle (Control) or 20 μΜ actinomycin D for 30 min, followed by addition of 50 μΜ NMDA for 15 min. Wnt5a protein was detected by Western blotting and quantified. The Graph is a summary of four independent experiments (*, p < 0.05; **, p < 0.01; One-way ANOVA). C. Primary cortical neurons (10 DIV) were treated with vehicle (Control) or 50 μΜ NMDA for 15 min. Wnt5a mRNA was quantified by Real-time RT-PCR (qPCR). The summary graph is from three independent experiments (40 cycles, CT values: 25.1 ± 0.5/control vs. 25.6 ± 0.3/NMDA; p > 0.05; two-tailed Student's tests). D. Melt curve of Wnt5a qPCR on control cells. The melt curve on NMDA-stimulated cells was similar (not shown). E. RT-PCR results of Wnt5a in control and NMDA-treated cells.

Given the importance of Wnt5a and NMDAR in the regulation of synaptic plasticity, we were interested in elucidating the mechanism by which NMDAR activation rapidly increases the intracellular Wnt5a concentration in cortical cultures. First, we tested the hypothesis that NMDAR activation caused Wnt5a increase by stimulating mRNA translation. To this end, we used the translation inhibitor, anisomycin (20 μM). We observed that pre-treatment of the cultures with anisomycin for 30 min before NMDA application completely abolished the Wnt5a increase elicited by NMDA stimulation (50 μM; 15 min) (Figure 2A). This result suggests that NMDAR activation stimulates Wnt5a production via *de novo *protein synthesis. Because mRNA translation is often coupled with gene transcription, we further tested the hypothesis that NMDARs up-regulate Wnt5a protein production via transcriptional activation. To this end, we used the transcription inhibitor, actinomycin D (25 μM). The cultures were pretreated with actinomycin D for 30 min before NMDA application. To our surprise, actinomycin D completely failed to block the Wnt5a increase (Figure 2B). In fact, actinomycin D appeared to increase Wnt5a in this short time window, which might be due to a stimulating effect of actinomycin D on translation [32]. This observation suggests that NMDARs evoke the rapid Wnt5a protein increase in a transcription-independent process. To verify this notion, we performed quantitative RT-PCR to compare Wnt5a mRNA levels in cultures with or without NMDA stimulation (15 min). No significant differences of Wnt5a mRNA levels were observed in control and treated cultures (Figure 2C, D). To confirm this observation, we also perform semi-quantitative RT-PCR. As shown in Figure 2E, no obvious difference was detected in the amount of the Wnt5a RT-PCR products from control and NMDA-stimulated cells. Collectively, results from this set of experiments suggest that NMDAR activation evokes rapid translation from pre-existing Wnt5a mRNA in neurons.

### mTOR signaling pathway is not required for the NMDAR-dependent Wnt5a protein synthesis

**Figure 3:**
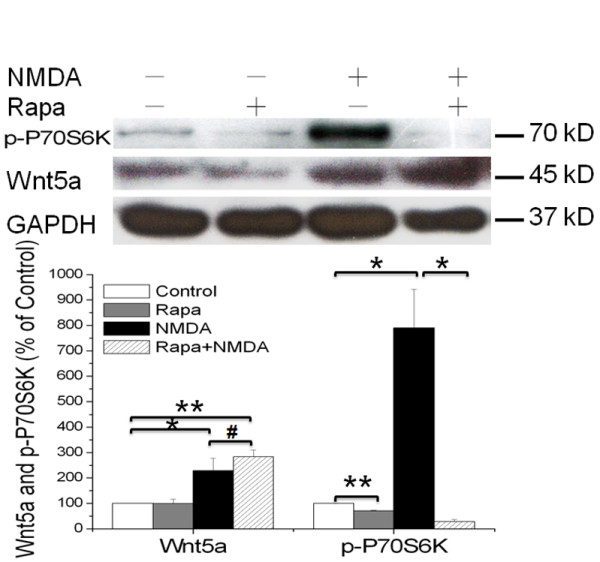
**mTOR signaling pathway is not required for the NMDAR-dependent Wnt5a protein synthesis**. Primary cortical neurons (10 DIV) were pre-treated with vehicle (Control) or 25nΜ Rapamycin for 30 min, followed by addition of 50 μΜ NMDA for 15 min. Western blotting analysis of Wnt5a and phosphor-P70S6K proteins were performed. Graphs (mean ± SEM) are from four independent experiments (*, p < 0.05; **, p < 0.01; #, p > 0.05; One-way ANOVA).

Previous studies have revealed that mTOR signaling is a major molecular pathway in the control of activity-regulated protein synthesis during synaptic plasticity [33-35]. The mTOR pathway is known to mediate NMDAR-dependent αCaMKII protein synthesis in hippocampal neurons [35]. And we have found that NMDAR stimulation induced phosphor-P70S6K (p-P70S6K, a downstream target of mTOR signaling) increase, this effect could be diminished by DAP5 (Figure 1C). Therefore, we tested the potential role of mTOR in NMDAR-induced Wnt5a translation. Interesting, we found that rapamycin (25 nM, 30 min of pretreatment), a specific inhibitor of mTOR kinase, did not affect NMDA-induced (50 μM; 15 min) Wnt5a protein increase (Figure 3). To rule out the possibility of experimental failures, we determined the effect of NMDA and rapamycin on the phosphorylation level of P70S6K. The results showed that NMDA treatment clearly increased p-P70S6K; this increase was abolished by rapamycin (Figure 3), indicating that NMDA activated mTOR signaling and that rapamycin was able to block this activation in our experimental systems. Thus, based on these results, we concluded that the NMDAR-dependent Wnt5a protein synthesis is not mediated by the mTOR signaling pathway.

### NMDAR activation stimulates Wnt5a protein synthesis via the MAPK signaling pathway

**Figure 4:**
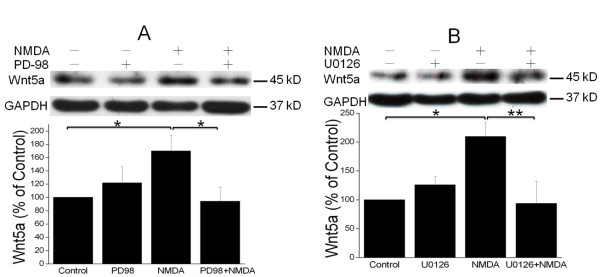
**NMDAR activation stimulates Wnt5a protein synthesis via the MAPK signaling pathway**. A. Primary cortical neurons (10 DIV) were pre-treated with vehicle (Control) or 20 μM PD98059 (PD98) for 30 min, and then stimulated with 50 μΜ NMDA for 15 min. Wnt5a protein was measured by Western blotting and quantified data were presented in graphs (mean ± SEM; n = 3, *p < 0.05; One-way ANOVA). B. Primary cortical neurons (10 DIV) were pre-treated with vehicle (Control) or 20 μM U0126 for 30 min, followed by 50 μΜ NMDA for 15 min. Graphs (mean ± SEM) are from three independent experiments (*, p < 0.05; **, p < 0.01; One-way ANOVA).

Previous studies indicate that MAPK signaling is critical for activity-regulated protein synthesis in neurons [36,37]. We investigated the involvement of MAPK signaling in NMDAR-dependent Wnt5a protein synthesis using pharmacological approaches. We observed that PD98059 (at 20 μM for 30 min pretreatment), a specific MEK inhibitor, blocked the NMDA-evoked Wnt5a increase (Figure 4A). To confirm this observation, we employed another MEK inhibitor, U0126, and we found that U0126 (20 μM, 30 min pretreatment) also diminished the NMDA-induced Wnt5a protein increase (Figure 4B). These findings strongly suggest that the MAPK signaling pathway is essential for NMDAR to activate Wnt5a translation.

## Conclusion and Discussion

In this study, we found that NMDAR activation rapidly increases the synthesis of Wnt5a protein. We further elucidate that the NMDAR-regulated rapid Wnt5a synthesis depends on translation but not transcription and that NMDAR-induced translation from the preexisting Wnt5a mRNA is activated by MAPK signaling but not the mTOR signaling pathway.

Inestrosa and co-workers showed that Wnt5a modulates the plasticity of both glutamatergic and GABAergic synapses on hippocampal neurons [20,21]. However, the mechanism of Wnt5a regulation during the induction and expression of synaptic plasticity was not known. Our findings reveal that synaptic activity, via NMDAR activation, stimulates the synthesis of Wnt5a protein. Because Wnt5a is in dendritic regions-near the presynaptic terminals in mature neurons (Figure 1A)-the rapid synthesis and secretion of Wnt5a following NMDAR activation probably provide an endogenous source of Wnt5a to alter the molecular organization and function of synapses. Indeed, Chen et al. reported that NMDAR-dependent secretion of Wnt3a regulates synaptic plasticity in hippocampal slices [7]. These findings collectively support the view that activity-regulated synthesis and secretion of Wnts are basic molecular processes underlying the expression of synaptic plasticity [8].

The increase in NMDAR-regulated Wnt5a protein is a result of *de novo *translation that does not require mRNA transcription (Figure 2A, C). These findings indicate that there is dormant Wnt5a mRNA stored in neurons, and this mRNA is positioned for translational initiation following NMDAR activation. This provides a mechanism for neurons to quickly generate new Wnt5a, which is probably needed for synaptic processes that are critical in the early stage of synaptic plasticity soon after synaptic activation, including the re-organization of synaptic proteins [20,21]. On the other hand, Wayman et al. showed that in differentiating hippocampal neurons NMDAR activation stimulates Wnt2 transcription, which regulates dendritic arborization [31]. Together, these findings indicate that NMDARs may evoke the expression of different Wnt proteins by stimulating either transcription or translation in different cellular contexts.

The mTOR signaling pathway is a key mechanism by which synaptic activity stimulates protein synthesis in neurons [38,39]. However, our results indicate that this pathway is not involved in the activation of NMDAR-regulated Wnt5a mRNA translation (Figure 4A, B). Instead, the NMDAR-elicited Wnt5a protein synthesis requires the activation of the MAPK signaling pathway. Tsokas et al. reported that MAPK signaling can stimulate activity-regulated synthesis of translational proteins by controlling the mTOR signaling pathway [40]. Because mTOR is not required for Wnt5a synthesis (Figure 3), we conclude that MAPK signaling leads to translational activation via an mTOR signaling-independent pathway.

**Figure 5:**
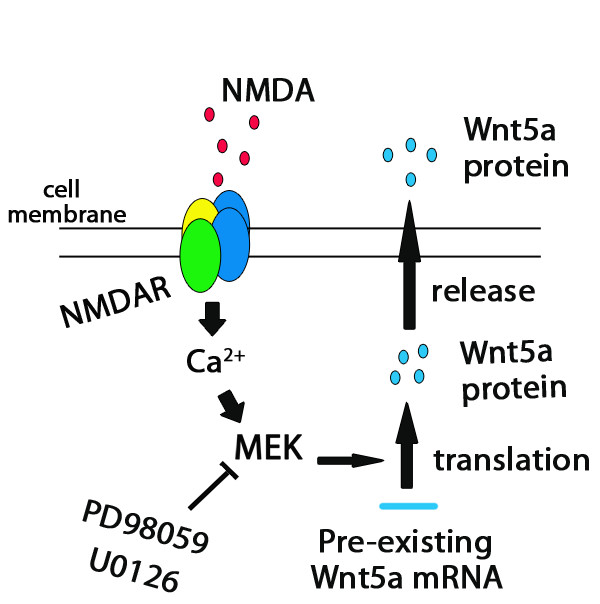
**A working model for NMDAR-regulated Wnt5a protein synthesis**. Stimulation of NMDAR activates MAPK signaling to elicit de novo translation from pre-existing Wnt5a mRNAs.

Based on the results presented here, we propose the following model: In resting neurons, Wnt5a mRNAs are stored in a translationally inactive form. When neurons are stimulated, synaptic activity induces Ca^2+ ^influx through NMDARs to activate MAPKs to elicit *de novo *Wnt5a mRNA translation (Figure 5).

## Materials and methods

### Compounds

NMDA [N-methyl-D-aspartic acid, product number (M3262)], DAP5 [D(-)-2-Amino-5-phosphonovaleric acid, A8054], Poly-D-lysine (P7280), U0126 (U120), Trypsin 10× solution (T4674), MSG (L-glutamic acid monosodium salt hydrate, G5889), Rapamycin (R8781), PD98059 (P215), Actinomycin D (A4262); Anisomycin (A9789) were purchased from Sigma; DAPI (s36939) from invitrogen; HBSS (Hank's balanced salt solution 10×, 14185), D-MEM/F-12 (Dulbecco's modified eagle medium: nutrient mixture F-12, 12400-024), L-Glutamine 100× (25030), B27 50×, NBM (Neurobasal medium, 21103) from Gibco; FBS (Fetal bovine serum, A15-101) from PAA; and DMSO (0231) from Amresco.

NMDA was dissolved in NBM 5 min before treatment. DAP5, U0126, Rapamycin, PD98059, Anisomycin were prepared as 1000× concentrated stocks in DMSO. All other compounds were prepared as 1000× concentrated stocks in ultrapure water.

### Antibodies

Anti-Wnt5a antibody was purchased from R&D Systems (AF654); anti-p-P70S6K (Thr389) antibody from Cell Signaling Technology (#9206); anti-GAPDH antibody from Santa Cruz (SC-32233); anti-Synapsin I from Millipore (AB1543P); and FITC-conjugated donkey anti-rabbit secondary antibody (711-097-020) and Rhodamine-conjugated rabbit anti-goat secondary antibody (305-297-003) from Jackson.

### Primary cortical culture

Cortical cultures were prepared as described (32). Briefly, cortices were dissected from C57BL/6J mouse embryos (E18) in HBSS, stripped from blood vessels, and cut into small pieces. They were then digested in 1× trypsin for 8 min at 37° in 5 ml tubes and dissociated into single cells by gentle aspirations with a fire-polished glass pipette. After sitting on the bench for 2 min, cells in the supernatant were transferred into fresh tubes and centrifuged for 5 min (1000 rpm). Cell pellets were suspended in DMEM. Cells were plated on 12-well plates (JETBIOFIL) with poly-D-lysine (20 μg/ml) at a density of 5 × 105 cells/well and incubated at 37° in a humidified atmosphere of 95% air and 5% CO2. One hour later, the culture media were replaced with NBM supplemented with 2% B27, 5 mM glutamine, 1% streptomycin and penicillin. The media were changed every three days. Cultures were used for stimulation at day 10 in vitro.

### Real-time fluorescence quantitative PCR

Cultures (10 DIV; 2 × 106 cells/well in 6-well plates) were switched to fresh media for 1 h and then stimulated with NMDA (50 μM) for 15 min at 37°. Total RNA was purified from the cultures with TRIZOL (invitrogen) according to the manufacturer's instructions. The RNA purity was determined by the OD260/OD280 ratio, and the concentration was calculated based on OD260. The RNA (1.5 μg) was used for reverse transcription, followed by quantitative real-time PCR using PrimeScriptTM RT reagent kit (TaKaRa). PCRs (25 μl reactions) contained 12.5 μl 2× SYBR Premix Ex TaqTM, 0.5 μl PCR Forward Primer (10 μM), 0.5 μl PCR Reverse Primer (10 μM), 9.5 μl dH2O and 2 μl cDNA. The following primers were employed: Wnt5a Reverse primer: 5'-AGCCAGCACGTCTTGAGGCTA-3'; Wnt5a Forward primer: 5'-AA TCCACGCTAAGGGTTCCTATGAG-3'; β-actin Reverse: 5'-GCAATGCCTGGGTACATGGTGG-3'; and β-actin Forward: 5'-ACGCGTCGACCTCCTTGCAGTCCATTTT-3'. PCR was run for one cycle at 95° for 10 s, and 40 cycles at 95° for 5 s; 60° for 20 s.

### Immunofluorescent staining

Primary cortical neurons that had been grown on glass coverslips were briefly washed twice with cold PBS, and then fixed in 4% paraformaldehyde for 30 min at room temperature (RT). Neurons after fixation were washed with cold PBS (3 × 5 min), permeabilized with 0.1% Triton X-100 for 10 min, rinsed three times, and blocked with 1% BSA in PBS for 1 h (RT). Next, neurons were incubated with primary antibodies (double-stained with anti-Wnt5a antibody at 1:50 and anti-synapsin I antibody at 1:200) in 1% BSA/PBS in a humidified chamber overnight at 4°, rinsed three times in PBS (3 × 5 min). This was followed by incubation with secondary antibodies [Rhodamine-conjugated anti rabbit IgG (recognizing anti-Wnt5a antibody) and FITC-conjugated anti goat IgG (recognizing anti-synapsin I antibody)] in 1% BSA/PBS in a light-proof container (1 h at RT). Then, cells were washed (3 × 5 min in PBS), stained with 0.1 μg/ml Hoechst for 1 min, and rinsed with PBS before being mounted.

### Western blotting

To detect intracellular proteins, cortical neurons in 12-well plates (5 × 105 cells/well; 10 DIV) were rinsed with PBS and lysed immediately in 100 μl of 2× SDS-PAGE sample buffer (1×: 62.5 mM Tris, pH 6.8, 2% SDS, 5% 2-mercaptoethanol, 10% glycerol, and 0.0025% bromophenol blue as described previously [32]). These were then boiled for 10 min. After electrophoresis on 10% SDS-PAGE gels, proteins were transferred to 0.2 μm Immobilon polyvinylidene difluoride (PVDF) membranes (Millipore) and blotted with primary and HRP-conjugated secondary antibodies. The signals were detected using the ECL system (Pierce). To detect secreted Wnt5a, media of cortical neurons in 12-well plates (1 × 106 cells/well; 10 DIV) were replaced with 300 μl NBM before NMDA stimulation. All NBM was collected after the stimulation and heat-evaporated to a final volume suitable for one loading on an SDS-PAGE gel.

### Quantification and statistics

Immunoblots were scanned with an Epson scanner, and the optical density (OD) of protein bands were quantified with Quantity One software (Bio-Rad). The statistical tests were performed by one-way ANOVA or by two-tailed Student's tests, using SPSS 16.0. Graphs of quantified data (Mean data + SEM) were prepared using Origin.

## Competing interests

The authors declare that they have no competing interests.

## Authors' contributions

YL and BL performed experiments, prepared the figures and drafted the manuscript. XW participated in a part of the study. XW provided critical reagents. LZ coordinated the performance of the study and contributed to experimental design and data analysis. SJT conceived of the study and participated in its design and manuscript writing. All authors read and approved the final manuscript.
